# Rosiglitazone and Pioglitazone Alter Aromatase Kinetic Properties in Human Granulosa Cells

**DOI:** 10.1155/2011/926438

**Published:** 2011-12-19

**Authors:** Takako Araki, Miroslava Varadinova, Michael Goldman, Zev Rosenwaks, Leonid Poretsky, Donna Seto-Young

**Affiliations:** ^1^Gerald J. Friedman Diabetes Institute and the Division of Endocrinology, Department of Medicine, Beth Israel Medical Center and Albert Einstein College of Medicine, New York, NY 10003, USA; ^2^Ronald O. Perelman and Cohen Center for Reproductive Medicine and Infertility, Weill Medical College of Cornell University, New York, NY 10021, USA

## Abstract

We have previously reported that, in human granulosa cells, thiazolidinediones rosiglitazone and pioglitazone inhibit estrogen synthesis by interfering with androgen binding to aromatase, without an effect on aromatase mRNA or protein expression. In the current paper, we explore the effects of rosiglitazone and pioglitazone on the aromatase enzyme kinetic properties in human granulosa cells. The cells were incubated with various concentrations of testosterone or androstenedione, with or without rosiglitazone or pioglitazone. Estradiol and estrone concentrations in the conditioned tissue culture medium were measured by radioimmunoassay or immunosorbent assay. When testosterone was used as substrate, rosiglitazone or pioglitazone inhibited the *V*_max_ by 35% (*P* < 0.001) and 24% (*P* < 0.001), respectively. When androstenedione was used as substrate, both rosiglitazone or pioglitazone inhibited *V*_max_ by 13% (*P* < 0.007). We conclude that rosiglitazone or pioglitazone has no effect on *K*_*m*_ but inhibits *V*_max_ of aromatase in human granulosa cells, therefore, acting as noncompetitive inhibitors.

## 1. Introduction

Thiazolidinediones (TZDs: troglitazone, rosiglitazone, and pioglitazone) are peroxisome proliferator-activated receptor-*γ* (PPAR-*γ*) agonists, which have been used in the treatment of type 2 diabetes as insulin sensitizers. TZDs have also been used as therapeutic agents for women with polycystic ovary syndrome (PCOS) since they reduce androgen levels and improve ovulatory rates [1–4]. Although the effects of TZDs in the female reproductive system have been attributed to their systemic insulin-sensitizing properties and consequent reduction in hyperinsulinemia [5], TZDs also directly affect androgen and estrogen production in human ovarian cells [6].

Aromatase is a cytochrome P450 superfamily enzyme that converts androgens to estrogens. The literature on the TZD effects on aromatase activity in the ovary is controversial [7–9]. Our previous studies demonstrated that rosiglitazone or pioglitazone directly inhibit estrogen synthesis in the mixed human ovarian cell culture containing stromal, thecal, and granulosa cells and in purified human granulosa cells obtained from *in vitro* fertilization [6, 10]. We recently reported that rosiglitazone or pioglitazone also interfere with androgen binding to aromatase enzyme, suggesting that TZDs may affect the aromatase enzyme kinetic properties [10].

The goal of this study, therefore, was to examine the effects of TZDs on the enzyme kinetic properties of aromatase.

## 2. Materials and Methods

The institutional review boards at the Beth Israel Medical Center and the Weill Medical College of Cornel University approved all studies described in this paper.

### 2.1. Human Granulosa Cells and Cell Culture

Human granulosa cells obtained during *in vitro* fertilization (IVF) were pooled from several patients at a time to ensure adequate cell number for the experiments. Diagnoses included male factor, tubal factor, and uterine factor infertility, endometriosis, or anovulation [10]. Human granulosa cells obtained during *in vitro* fertilization were purified on the Percoll gradients (50% Percoll/Hank's balanced salt solution) as previously described [10]. Purified human granulosa cells were counted using hemocytometer, and 1 mL of 0.5 × 10^5^ cells/mL suspension was placed in 24 well tissue culture plates [10]. The cells were cultured for 48 hours at 37°C, 5% CO_2_, 90% humidity in M199 medium supplemented with 10% FBS, 10 *μ*g/mL gentamicin, and 250 ng/mL amphotericin B. After 48 hours of incubation, the medium supplemented with 10% FBS was replaced by a medium with 2% FBS, in which the cells were incubated for additional 24 h before the kinetic experiments were performed.

### 2.2. Enzyme Kinetic Studies

For the aromatase enzyme kinetic studies, cells were incubated with various concentrations of testosterone (0.025, 0.05, 0.1, 0.133, 0.25, or 1.0 *μ*M) or androstenedione (0.025, 0.05, 0.1, 0.133, 0.25, 0.5, or 1.0 *μ*M) for 150 min, in the presence or absence of 25 *μ*M rosiglitazone or pioglitazone. The conditioned tissue culture medium was collected for determination of estrone and estradiol concentrations. Several time points (150 min, 240 min, 360 min, and 720 min) were included in the kinetic experiments. The velocity of aromatase activity (*μ*M estradiol/mg/min) was 0.14, 0.25, 0.22, and 0.28, respectively, for the 150, 240, 360, and 720 min of the reaction time points. Because 150 min incubation time produced a linear reaction, this time point was chosen for the kinetic studies. The concentrations of the substrates were extended to those below and above the *K*_*m*_.

### 2.3. Protein Measurement

Protein concentrations were determined by BCA (Bicinchoninic Acid Assay) [11].

### 2.4. Radioimmunoassay and Immunosorbent Assay

Estrone concentrations in the tissue culture medium were measured using enzyme-linked immunosorbent assay (ELISA). The cross-reactivity with estrone is 100%, with estradiol 2.2%, and with other related compounds 0.14%. Estradiol concentration was measured using radioimmunoassay (RIA). The cross-reactivity with 17*β*-estradiol is 100% and with other related compounds less than 1%.

### 2.5. Materials

M199 medium, heat-inactivated fetal bovine serum (FBS), gentamicin, and amphotericin were obtained from Invitrogen Corp., Carlsbad, CA, USA; testosterone and androstenedione were from Sigma-Aldrich Corp., St. Louis, Mo; rosiglitazone was from Cayman Chemical, Ann Arbor, Mich; pioglitazone was from Takeda Pharmaceuticals America, Inc. Lincolnshire, IL; Lowry protein assay kits were from Thermo Scientific, Rockford, IL, USA; estrone enzyme-linked immuno-sorbent assay (ELISA) kits were from Alpco Diagnostics, Salem, NH; estradiol radioimmunoassay (RIA) kits were from the Diagnostic Systems Laboratories, Webster, TX, USA.

### 2.6. Statistical Analysis

All experiments were carried out in triplicates or quadruplicates and repeated 6 to 7 times. Two-way analysis of variance (ANOVA) was used to compare mean values according to substrate concentrations (testosterone or androstenedione) in the presence or absence of rosiglitazone or pioglitazone. The statistical interactions between the sets of data obtained with or without rosiglitazone or pioglitazone were examined. Pairwise Bonferroni's adjusted contrasts were used to determine statistical significance.

## 3. Results

### 3.1. Testosterone as Substrate

**Figure 1:**
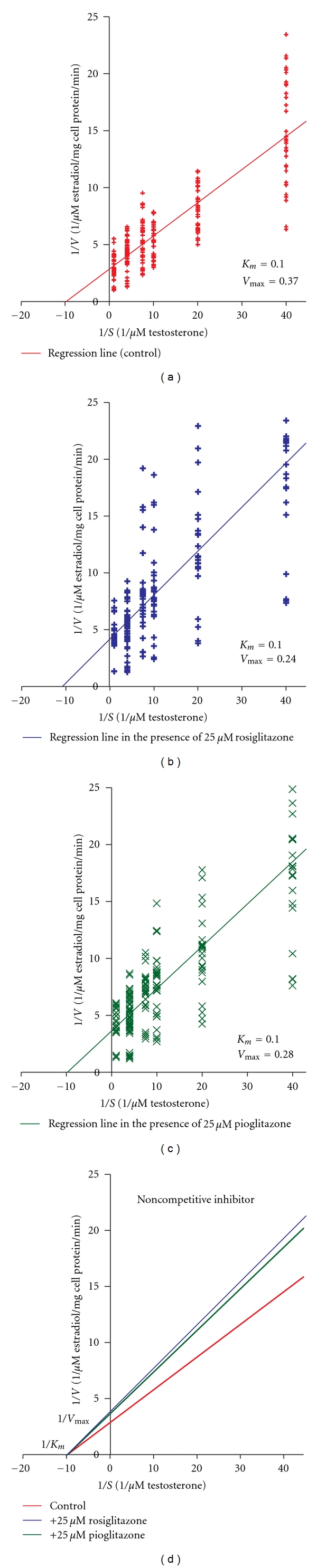
Effects of TZDs on aromatase enzyme kinetics when testosterone was used as substrate. (a) The Lineweaver-Burk plot (reciprocal velocities of aromatase activity against reciprocal testosterone concentration) in the absence of rosiglitazone or pioglitazone. (b) The Lineweaver-Burk plot in the presence of rosiglitazone. (c) The Lineweaver-Burk plot in the presence of pioglitazone. (d) Comparison of the Lineweaver-Burk plots in the presence or absence of rosiglitazone or pioglitazone. *V* = velocity; *S* = substrate.

When testosterone was used as substrate, aromatase *V*_max⁡_ was reduced by 35% with rosiglitazone (*P* < 0.001) (Figures 1(a) and 1(b)) and by 24% with pioglitazone (*P* < 0.001) (Figures 1(a) and 1(c)). The *K*_*m*_ was not affected (Figure 1(d)).

### 3.2. Androstenedione as Substrate

**Figure 2:**
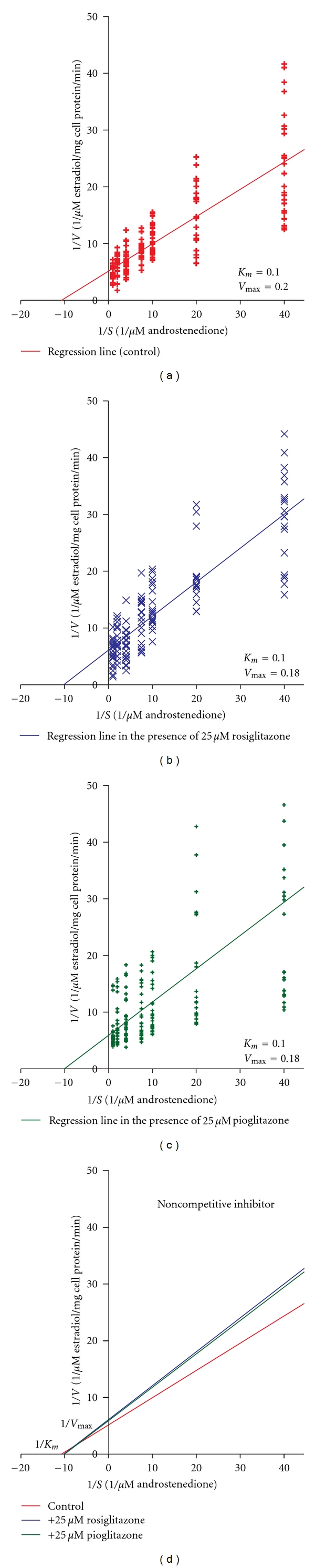
Effects of TZDs on aromatase enzyme kinetics when androstenedione was used as substrate. (a) The Lineweaver-Burk plot (reciprocal velocities of aromatase activity against reciprocal androstenedione concentration) in the absence of rosiglitazone or pioglitazone. (b) The Lineweaver-Burk plot in the presence of rosiglitazone. (c) The Lineweaver-Burk plot in the presence of pioglitazone. (d) Comparison of the Lineweaver-Burk plots in the presence or absence of rosiglitazone or pioglitazone. *V* = velocity; *S* = substrate.

When androstenedione was used as substrate, rosiglitazone inhibited *V*_max⁡_ by 13% (*P* < 0.007) (Figures 2(a) and 2(b)). Pioglitazone also inhibited *V*_max⁡_ by 13% (*P* < 0.004) (Figures 2(a) and 2(c)). Neither rosiglitazone nor pioglitazone had significant effect on *K*_*m*_ of aromatase (Figure 2(d)).

## 4. Discussion

Aromatase is a cytochrome P450 superfamily enzyme that converts androgens to estrogens and is highly expressed in granulosa cells. It is a key enzyme for the development of sexual characteristics, and its function is important in the pathogenesis of various diseases, including breast cancer [12–14].

Conflicting reports have been published regarding the effects of TZDs on the expression and activity of aromatase in the ovary [8, 9, 15]. For example, Gasic et al. reported absence of troglitazone effects on the aromatase in porcine granulosa cells [15], while other studies reported that troglitazone/LG100268 induced suppression of enzymatic expression and activity of aromatase in human granulosa cells and in granulosa cell carcinoma cell lines [8, 9]. In our previous studies, rosiglitazone or pioglitazone directly inhibited estrogen synthesis in human mixed ovarian cell culture containing stromal, thecal, and granulosa cells [6]. Recently we reported that the mechanism of rosiglitazone or pioglitazone effect on estrogen synthesis in cultured human granulosa cells does not involve any effects on either aromatase mRNA or protein expression [10]. In contrast, another thiazolidinedione, troglitazone, inhibited aromatase mRNA expression in our studies, which is consistent with the literature [8, 9]. We also showed that rosiglitazone or pioglitazone inhibited androgen binding to aromatase [10].

In this study, we examined the effects of rosiglitazone or pioglitazone on aromatase enzyme kinetic properties. Rosiglitazone or pioglitazone inhibited *V*_max⁡_ of aromatase without affecting the *K*_*m*_, suggesting that rosiglitazone or pioglitazone inhibit aromatase enzyme in a noncompetitive manner (rosiglitazone or pioglitazone did not compete with substrate—testosterone or androstenedione—binding to aromatase). Our previous studies examined ^125^[I]-testosterone or ^125^[I]-androstenedione binding to immuno-purified aromatase bound to protein A immobilized on agarose [10]. Iodination of the substrates and procedure used for immunopurification of aromatase may have altered the substrate and enzyme structure in these studies. The current studies, which involved assessing initial activity rate of aromatase in the presence of various concentrations of substrates, allowed us to obtain more accurate Michaelis-Menten constants *K*_*m*_ and *V*_max⁡_.

The competitive aromatase inhibitors (anastrozole and letrozole) used in clinical practice to treat or to prevent breast cancer inhibit aromatase activity by 90–100%. Rosiglitazone and pioglitazone, in contrast, inhibit aromatase activity by 30 to 50% [10]. The current studies show a 13–35% reduction in aromatase activity because the substrate concentrations were between 0.025 and 1 *μ*M, which are either below or just above the *K*_*m*_. In the previous studies, the substrate concentrations were above the *K*_*m*_ (3 *μ*M), and the incubation time was 18 hours.

This difference in potency may make thiazolidinediones an attractive alternative to competitive aromatase inhibitors because of presumably lower likelihood of aromatase-related side effects with TZDs (such as hot flushes and reduced bone density). In healthy postmenopausal women, annual bone loss associated with traditional aromatase inhibitors is higher than that observed with TZDs (2.2–2.6% versus 0.5%, resp.). Inhibition of estrogen synthesis by TZDs may make them useful in the therapy of estrogen-dependent diseases including breast cancer, gynecomastia, uterine fibroids, and endometrial cancer [12, 13]. Inhibition of aromatase activity by TZDs may also help explain their negative effect on bone density in older or postmenopausal women [16, 17].

In conclusion, rosiglitazone or pioglitazone inhibits the aromatase enzyme activity by reducing its *V*_max⁡_ without effect on *K*_*m*_, acting as noncompetitive inhibitors of the aromatase enzyme.
